# Supplementary material for: Revisiting the physical limits to economic growth, with a focus on the waste heat limit
Source: PLoS One. 2025 Mar 13;20(3):e0319217. doi: 10.1371/journal.pone.0319217 (PMC11906051; doi:10.1371/journal.pone.0319217)
Supplement: S1 Table — Notes. Table provides estimates of historical factor shares, estimated using Eq (7). Specifically, it presents results of a regression of the growth rate of GDP per employee on the growth rate of non-power physical capital per employee and on the growth rate of power per employee. The coefficient on the former regressor yields an estimate of θK and the latter an estimate of θP. The constant provides an estimate of the average growth rate of TFP (gA). Data are from Penn World Tables (PWT) 10 and Our World in Data (OWID). The sample includes countries with both (a) income, employee population, and capital stock and (b) power consumption for the entire period from 1965–2019. Data are available for 66 countries from 1965–1991; in 1992 USSR is replaced with 14 countries that replaced the USSR. Moldova is excluded due to lack of data. (PDF) [file pone.0319217.s001.pdf]

S1 Table. Estimating factor shares from historical, country-year level data from 1965-2019.

	Growth rate income/employee
Constant	0.0095
	(0.0015)
Capital/employee growth rate	0.5381
	(0.0338)
Power/employee growth rate	0.0199
	(0.0048)
Observations	3788

Notes. Table provides estimates of historical factor shares, estimated using Eq. (7). Specifically, it presents results of a regression of the growth rate of GDP per employee on the growth rate of non-power physical capital per employee and on the growth rate of power per employee. The coefficient on the former regressor yields an estimate of θ_K and the latter an estimate of θ_P. The constant provides an estimate of the average growth rate of TFP (g_A). Data are from Penn World Tables (PWT) 10 and Our World in Data (OWID). The sample includes countries with both (a) income, employee population, and capital stock and (b) power consumption for the entire period from 1965-2019. Data are available for 66 countries from 1965-1991; in 1992 USSR is replaced with 14 countries that replaced the USSR. Moldova is excluded due to lack of data.
