# Supplementary material for: Revisiting the physical limits to economic growth, with a focus on the waste heat limit
Source: PLoS One. 2025 Mar 13;20(3):e0319217. doi: 10.1371/journal.pone.0319217 (PMC11906051; doi:10.1371/journal.pone.0319217)
Supplement: S1 Table — Notes. Table provides geometric means of growth rates of per capita income, population, capital stock, and power consumption for 92 countries from 2000-2018 using PWT data. These means are calculated at the country x year level, where countries are weighted by their GDP. 2019 is omitted because we do not have data for 2020 and thus growth rates for 2019. Units are fraction or %/100. These means are used, along with Eqs (8) and (9), to forecast when the waste heat limit will occur in Figs 2 and 3. (PDF) [file pone.0319217.s002.pdf]

S2 Table. Average growth rates of key parameters for 92 countries form 2000-2018.

	Geometric mean	[95% conf.	interval]
Per capita output	0.0385	0.0370	0.0400
Output	0.0471	0.0456	0.0487
Population	0.0114	0.0111	0.0117
Employed pop.	0.0136	0.0131	0.0141
Capital stock	0.0508	0.0496	0.0521
Power	0.0402	0.0388	0.0417
TFP	0.0130	.	.

Notes. Table provides geometric means of growth rates of per capita income, population, capital stock, and power consumption for 92 countries from 2000-2018 using PWT data. These means are calculated at the country x year level, where countries are weighted by their GDP. 2019 is omitted because we do not have data for 2020 and thus growth rates for 2019. Units are fraction or %/100. These means are used, along with Eq. (8) and Eq. (9), to forecast when the waste heat limit will occur in Figures 2 and 3.
