# Supplementary material for: Revisiting the physical limits to economic growth, with a focus on the waste heat limit
Source: PLoS One. 2025 Mar 13;20(3):e0319217. doi: 10.1371/journal.pone.0319217 (PMC11906051; doi:10.1371/journal.pone.0319217)
Supplement: S3 Table — Notes. Table presents the 2019 values of total employment, total population, total primary power consumption, and average TFP for 222 countries. We use these as initial values for the plots in Fig 2. Total employees, population, and power consumption is obtained for all 222 countries for which these data are available in 2019 from PWT 10.0 and Our World in Data. We obtain average TFP in 2 steps. First, we take estimates of factor shares from S1 Table and use them to estimate TFP using Eq (5) for each of the 222 countries in 2019. Second, we take an arithmetic average of those TFPs. (PDF) [file pone.0319217.s003.pdf]

S3 Table. Initial values from 2019 for future projections.

	2019 values
Countries (no.)	222
Employed workers (billions)	3.316
Population (billions)	7.581
Primary energy consumption (TwH)	163231
Average TFP ($USD/workers^{\theta\_N} \cdot TwH^{\theta\_P}$	51.5
Geometric mean TFP ($USD/workers^{\theta\_N} \cdot TwH^{\theta\_P}$	46.85912

Notes. Table presents the 2019 values of total employment, total population, total primary power consumption, and average TFP for 222 countries. We use these as initial values for the plots in Figure 2. Total employees, population, and power consumption is obtained for all 222 countries for which these data are available in 2019 from PWT 10.0 and Our World in Data. We obtain average TFP in 2 steps. First, we take estimates of factor shares from tab:histFactShares and use them to estimate TFP using Eq. (5) for each of the 222 countries in 2019. Second, we take an arithmetic average of those TFPs.
