# Supplementary material for: Revisiting the physical limits to economic growth, with a focus on the waste heat limit
Source: PLoS One. 2025 Mar 13;20(3):e0319217. doi: 10.1371/journal.pone.0319217 (PMC11906051; doi:10.1371/journal.pone.0319217)
Supplement: Attachment — Submitted filename: Response to Reviewers.pdf [file pone.0319217.s004.pdf]

Dear Dr. Boulanouar,

We thank you and the reviewers for helpful suggestions and a fruitful review process. We have addressed the journal requirements you highlighted. In response to the reviewers' helpful suggestions, we have made the following major revisions:

1. We made clear in the abstract and the introduction that there are two positions in the debate over the limits to growth, and while natural scientists and economists tend to be on opposite sides, that is not always the case.
2. We modified the text on line 162 to cabin one of our claims in response commnet #3 from referee #1.
3. We limited references to “indefinite growth” to address a referee's concern that there will be physical limits at some point unless all growth becomes fully non-physical.
4. On line 532, we added a paragraph to address concerns about our functional form assumptions for our production functions.
5. To further address concerns about limits to growth other than power, we added an additional short discussion on other limits to growth on line 65.
6. We added additional explanation on how we constructed Figure 2 on line 356.

Below please find our detailed responses to the reviewer's comments (in bold). Thank you again for handling our paper. We look forward to your response.

Sincerely,
Dorian S. Abbot and Anup Malani

Journal Requirements:

When submitting your revision, we need you to address these additional requirements.

1. Please ensure that your manuscript meets PLOS ONE's style requirements, including those for file naming. The PLOS ONE style templates can be found at https://journals.plos.org/plosone/s/file?id=wjVg/PLOSOne_formatting_sample_main_body.pdf and https://journals.plos.org/plosone/s/file?id=ba62/PLOSOne_formatting_sample_title_authors_affiliations.pdf

> Our submission used the PLOS One latex template here: https://journals.plos.org/plosone/s/latex. We hope that is sufficient.

2. Please include a caption for figure 4.

> We include the following caption:
>
> > **Use of 18 different commodities, relative to use of power, over time.** Legends indicate specific commodities. Ratio is units of specific commodity divided by units of power, with the ratio normalized to be 1 in 1965. Data are from the US Geological Survey.

3. We notice that your supplementary tables are included in the manuscript file. Please remove them and upload them with the file type 'Supporting Information'. Please ensure that each Supporting Information file has a legend listed in the manuscript after the references list.

> We moved the supplementary tables and listed titles and legends after the references list.

Reviewer #1:

Comment #1: This study assessed the impact of waste heat from electricity consumption on the Earth's surface temperature. I could not understand why waste heat from direct combustion of fossil fuels was not considered. Please explain the reason.

The term P_0 includes both power and heat required to produce it. We have added an explanation of this to the text (line 186): "(and waste heat required to generate and use that power)"

Comment #2 (page 5, lines 141-161): The authors analyzed the relationships between electricity consumption, population, per capita income, and energy efficiency using time series data from 1965 to 2020. By comparing the average growth rates of the socioeconomic variables, the authors found that population and economic growth were possible without growth in electricity consumption. I am concerned that this result depends on the choice of data period. Is the authors' finding robust to changes in the data period?

We use the entire period because that is the range for which we have data. However, to address the referee's concerns, we have calculated growth rates for power, population, per capita GDP, and implied average productivity growth for each 5 year period between 1965-2020. See below. The growth rate for each variable varies over time. However, the median growth rate across periods are not dramatically different than the 2.3% (g_P, power), 1.54% (g_N, population), 1.73% (g_y, economic growth), and the implied 1.2% (g_AP, average productivity) growth from 1965-2020. Also, notice that g_AP is positive for all sub-periods, demonstrating that population and economic growth are possible in them without growth in power consumption.

	g_P	g_N	g_y	g_AP
1965-1969	4.79	2.09	2.96	0.26
1970-1974	2.69	1.98	1.71	1
1975-1979	2.64	1.77	2.11	1.24
1980-1984	1.16	1.77	0.86	1.47
1985-1989	2.26	1.76	1.86	1.36
1990-1994	0.97	1.59	0.73	1.35
1995-1999	1.68	1.42	2.22	1.96
2000-2004	2.74	1.3	1.86	0.42
2005-2009	1.82	1.24	1.53	0.95

2010-2014	1.24	1.22	1.76	1.74
2015-2019	0.19	1.1	0.67	1.58
Median	1.82	1.59	1.76	1.35

Comment #3 (page 5, Figure 1): The plot of gP, gN, gy, and gAp may be more appropriate for the context of the manuscript than the plot of P, N, y, and Ap.

We have modified Figure 1 to show both P, N, y and Ap (in blue) and gP, gN, gy, and gAp (in red).

Comment #4 (page 5, lines 141-161): The manuscript says: "Evidently, current increases in efficiency are capable of driving significant economic and population growth even in the absence of growth in power consumption." This statement seems odd. Equation (2) is an identity and always holds mathematically. However, it does not mean that we can freely choose a combination (gN, gy, gAp) that satisfies 0 = gN + gy - gAp (How can we control the growth rates?). The above statement is mathematically true, but may not be feasible in the real world.

We have modified the text you quoted to explain your objection and to cabin our claim. The new text (line 162) reads "Evidently, if historical increases in efficiency continue, they will be capable of driving significant economic and population growth even in the absence of growth in power consumption. Of course one cannot ``choose'' future growth in power usage, economic growth, and population growth. But this analysis suggests it is physically possible to have reasonable population and economic growth---even if power growth is contained--if historical progress in efficiency continues."

Comment #5 (pages 7-8, lines 271-291): The authors estimated the parameters of the production function (Equation (5)) using panel data for many countries. However, the differences in the parameters across countries are not examined in this study. Is there any reason why the authors used country data for parameter estimation instead of aggregated global data?

We considered both. Ultimately, we chose to work with country-level data for two reasons. First, the economics literature works with country-level rather than global data. Each country is thought to have its own production function because an important contribution to the returns from capital and labor is the productivity term A, which includes things like market structure and regulation, features that vary substantially across countries. Global output is, identically, the sum of country level output. Second, there is more data at the country level and so our estimates of thetas were more precise when we exploit not just time series variation, but also cross-sectional variation in input and outputs.

Comment #6 (page 8): The authors should show that the TFP growth model expressed in Equation (7) is able to explain historical changes in TFP. Please estimate the parameters of the TFP growth model using historical data and summarize the result in the SI.

We attempted to do this but were unsuccessful because the estimation equation is not robust (sensitive to small changes in specification) for two reasons. First, we do not observe A(t). Instead we calculate A(t) at the country level using the factor shares (theta's) estimated with OLS from equation (6) and report it in Table S1 and plugin those into the log of eq. (5), i.e., A(i,t) for country i = log(Y(i,t)/L(i,t))L - theta_K (K(i,t)/L(i,t)) - theta_P (P(i,t)_/L(i,t)), where i indexes countries. Because this is a derived estimate, it has sampling variation. Second, eq. (7) is a function of growth in this estimate of A(i,t), and small variation in this can cause massive jumps in growth rates, especially for countries with low levels of productivity. We see this in our data: there are many growth rates, even when absolute differences in productivity are not large. As a result, when we try to directly estimate eq. (7), we get very low R^2 and even negative estimates of \lambda (i.e., negative return of population on productivity).

So, instead of estimating \lambda and \beta directly, we do two things. First, we mention estimates from the literature, specifically Bloom et al. (28) on line 336, which obtained micro-level information on a range of industries to estimate \beta for different \lambda's. Second, and most importantly, we showed how estimates of time to heat death vary with \lambda and \beta in Figs. 2 and 3. Indeed, this is critical to our central conclusion that we need better estimates of these 2 parameters, as well as factor shares, in order to make any reasonable guess as to the time until heat death, contra Murphy and others who predict limits to growth relatively soon.

Reviewer #2:

First of all, the authors pit natural scientists against economists, whereas there is also disagreement between economists on this issue. Secondly, they caricature the debate between the two camps, suggesting that the first camp ignores productivity improvements and substitution possibilities (between natural and man-made inputs). In the contemporary view, the proponents of possible limits to growth do not deny either of these possibilities but put forward arguments, based on the laws of physics, to explain why these two types of possibilities are limited. The authors never refer to these arguments. Thereby, the proposed framework seems hardly adequate to resolve the disagreement it seeks to resolve.

We respectfully disagree that the paper pits natural scientists against economists. First, the Abstract and Author Summary in the original draft said, "Whether there are imminent physical limits to growth has been the focus of a long-standing debate, often pitting natural scientists against economists." We have modified this to insert "(but not exclusively)" after "often" (see e.g., line 3). This is a fairly limited claim that often these two groups argue on opposite sides.

Second, we believe this limited claim is true. It is certainly correct that natural scientists — post Malthus, who was a reverend with economic ideas — were the ones who led the limits to growth debate (see lines 4-24). While some economists may have agreed, they were in the minority both among economists and among those who agree with the "limits to growth" argument. This remains true in the modern debate after the explosion of world population in the 1900s. Meanwhile, economists, led by people like Julian Simon – but not him alone, were the main group to disagree with the limits to growth argument. Characteristically, one of the more recent, well-argued limits to growth claims comes from a physicist (Murphy [5]). And among skeptics of limits to growth, economists tend to be the ones providing theory and data to support their claims.

While it is true that research on global warming has caused some economists to move to the limits to growth side of the debate, it is again a minority of economists and those economists are a minority of scholars in the limits to growth camp. Among economists, the dominant view remains that humans should not slow growth in light of climate change, but instead will innovate (e.g., Paul Romer) and adapt (e.g., Matthew Kahn).

All this said, we do think that we could do more to clarify our position – or at least make sure people do not think that the limits to growth debate is simply a debate among two academic disciplines. Therefore, in our Abstract and Author Summary, we clarify that the two sides that argue are the two sides of the debate, not the two disciplines, by noting the "One side <u>of the debate</u> posits that finite resources constrain human economic growth, the other that productivity improvements and substitution can relax those resource constraints," where the underlined text is new.

Moreover, for the main part of their paper, 1) the authors make the choice of a technological assumption (Cobb Douglas technology), which is known to be inconsistent with the implications of the law of physics (see e.g. Baumgärtner (2004), Energy and Resource Economics, Meran (2019), Energy Economics); 2) they assume that technical progress is a process that only requires labour (see equation (7)) and does not consume any resource (here power).

The combination of such types of assumptions is known to be likely to produce more favourable results to growth since assumption (1) amounts to assuming that the marginal productivity of resources is unbounded and assumption (2) implies that there is no limit to technological progress. By this same token, the article unsurprisingly leads to rather comforting results about our growth prospects.

Before answering this important comment, we should clarify what our paper contends.

- It does not contend that there exists a stable growth equilibrium path that lasts forever. Rather it contends that the limits to growth argument is very sensitive to parameter choices (Abstract: "We find remarkable parameter sensitivity: across a plausible range of parameters waste heat either never constrains economic growth in the model or does so in a few hundred years.") and it contends that economic growth will continue *but not be unbounded* (Abstract: "[O]ur work highlights the potential for an optimistic future for humanity in which economic growth continues on a millennial timescale.")

- Importantly, on this last point, note that our penultimate paragraph (in the Discussion section) says:

  > "Although our analysis suggests that physical limits to growth are both highly sensitive to critical parameter values and potentially far off in the future, there remains merit to the limits to growth argument for some variables on long enough timescales. For example, at a population growth rate of 1%/yr the mass of humans would equal the mass of Earth in approximately 3000 years and the mass of the observable universe in approximately 9000 years. Moreover, given that a human has a power output of ~100~W, the power output of humanity would hit the waste heat limit in 1400 years at a population growth rate of 1%/yr. So population is physically limited in a way that per capita income is not. That said, this point should not be taken as requiring immediate restrictions on economic and population growth."

- Moreover, in response to the referee's concern, we have further caveated our projections. In a few places, we had noted models might predict indefinite growth. In those places we have endeavored to replace "indefinite future" with

“distant future” (line 236) or “indefinitely extend” with “dramatically extend” (see abstract)

The fact that our manuscript is arguing about the *timescale for limits to growth* rather than the *existence of limits to growth* means that the results in papers such as Baumgartner and Meran are not at tension with our paper.

Baumgartner shows that if there exists some resource R that is necessary for production in the sense that Y = F(K,L,R) and the mass fraction of R in Y is \rho (fixed), then the first law of thermodynamics means that there must be an upper bound on the marginal productivity and average product of that resource, in violation of the Inada condition that lim_{R->\infty} \partial F/\partial R = 0. (Put aside the fact that the debate over dematerialization is whether \rho is actually fixed—even though economists dispute that \rho is fixed++. We will take Baumgartner’s claim at face value as his backup claim in Section 4 of his article is that not all consumption uses no resources.) The problem is that the Inada condition is required to prove the existence of a steady state growth path that lasts *forever*. We do not contend our argument or evidence shows that growth can last forever. Instead our manuscript is about how long it can last, with both limits to growth advocates and the manuscript agreeing the limit will be reached in finite time with unending growth. Indeed, Baumgartner does not make any argument about *when* the limit to growth will be reached, but rather that the limit will someday be reached.

Our response to Meran (2019) is similar. Meran demonstrates that the CES function, on its own, does not comply with the second law of thermodynamics. Moran’s solution is that one can impose a law of thermodynamics constraint on CES or come up with a production function that does satisfy thermodynamic laws. We have no problem with imposing a constrained CES or with constrained production in general, as our question is the timescale of when they apply, if they must apply. Meran has nothing to say about this as the constraint (eq. 9 in Meran) depends on parameters not given a priori (and not met yet). Moreover, Meran ignores the substitution question that Baumgartner addressed, a substitute which itself is not given a time frame.

Finally, it is worth addressing the core of the referee’s question: why do we use Cobb-Douglas production? First, we use CES in the substitution section. Second, substituting in CES for CD in the section on parameter sensitivity would not change our conclusions about parameter sensitivity, but would be more complicated to work with. We chose the simplest model to illustrate our point. The fact that Meran says even CES violates thermodynamic laws means that a switch to CES would not eliminate the referee’s criticism. What does address that criticism is that our inquiry does not require us to deny those limits. Again, our main argument is about when those limits are reached.

In response to the referee’s important comment, we have added a paragraph to the discussion clarifying our points above (see line 532):

“The functional form assumptions we make when conducting our simulation exercises do not qualitatively undermine our conclusion about the uncertainty about when limits to growth will bind. For example, our analysis employs production functions -- Cobb-Douglas (CD) or constant-elasticity-of-substitution (CES) -- that Baumgärtner \cite{Baumgärtner2004inada}, Meran \cite{Meran2019CES} and others have shown do not obey the laws of thermodynamics. However, use of the alternative production functions, including CES with a thermodynamic constraint, recommended \cite{Meran2019CES} as substitutes would yield qualitatively similar results, i.e., substantial sensitivity to critical parameter values. Moreover, we neither assume or conclude that there are no limits to growth. Rather, our view is that the existence of asymptotic limits on economic models does not imply that these constraints are imminent or require immediate curbs on economic activity. It is possible (but not certain) that physical limits to economic growth are many hundreds or thousands of years in the future.”

++To illustrate the economist position, consider an example for fixed \rho in Baumgartner:

“For instance, in order to produce 1 kg of iron screws one needs to employ at least 1 kg of pure iron. This means that \rho is physically bounded from below, in particular \rho > 0. Therefore, one may take q as a constant denoting the lower bound to dematerialization, i.e. \rho = const. with 0 < \rho < 1 denotes the minimum (mass) fraction of resource material contained in the output.”

The main objection economists would have to this is that it implicitly assumes that iron screws are required to produce goods or services that humans desire. Individuals may change what they consume, shifting towards consumption of e.g., services, as goods requiring material inputs increase in price as we approach \rho.

It is however fair to say that some results of the sensitivity analysis proposed by the authors are less reassuring: when beta is below 1, i.e. when technical progress is less easy, things get more complicated. This is precisely one of the 2 points I raised above...

We agree. This is precisely what we highlight in the full paragraph beginning in line 371. Recall, the punchline of our paper is that there is extreme parameter sensitivity that requires more investigation.

For the main part of their paper, the authors are only interested in one type of resource: "power". No distinction is e.g. made between energy and non energy resource. They do not motivate why restricting theirselves to a single dimension is enough to offer a right framework for the ambitious goal they announce.

First, our paper is motivated by a long line of work on the limits to growth, but it takes as a starting point a recent, strong contribution to that literature: Murphy (2022). And Murphy uses power as its hook. It also has the advantage of being amenable to formal modeling in a manner that permits exploration of different relevant themes (population growth, productivity growth, importance of parametric assumptions).

Second, we understand that power is not the only constraint. First, eqn 5 in the section “Future productivity and limits to growth” acknowledges there are other inputs, even as that section focuses on power, as an example. Second, the entire section, “Growth due to substitution to other resources” focuses on other constraints. Indeed, its first two sentences (line 422) read: “One might object that inputs into production of power are but one set of limited resources. Even if limits on power turn out not to constrain growth, perhaps other resources could.”

The status of the section entitled ``Growth due to substitution to other resources" from lines 406 to 493 is unclear to me even though it implicitly aims at answering to my previous point of criticism. But methodologically, this section is not really linked to the approach of the previous ones. By using a multi-input technology, they want to broaden the range of resources they take into account, whereas until now they have only talked about power. It is a sort of appendix in which the authors confirm that in the past there have been substantial changes in the input mix used. This shows that there has been a good "degree of plasticity in technology". I have no difficulty with that claim based on past evidence. But after this observation, the section suddenly ends with a profession of faith: ``this technological plasticity inspires confidence in humankind's ability to adapt to resource limits without sacrificing economic growth...". This statement is a conviction and not something that the section demonstrates. This section does not even explicitly address the question of resource limits.

The referee’s main objection appears to be that because something is true historically does not mean it is true in the future. We agree with the statement. Indeed, on line 504 (the sentence before the quoted text), we say:

> Past is not necessarily prologue, as suggested by the non-monotonicity in resource ratios in Fig. 4.

However, we disagree with the implications of the referee’s point about history.

First, the referee’s argument proves too much. In the past, we have experienced limits to growth (e.g., the entire period from the Neolithic Revolution to the dawn of the Industrial Revolution), limits that – along with ecological models of animals – inspire Malthusian models behind limits to growth. But we would not think it a sufficient argument against limits to growth to be that past limits do not imply future limits.

Second, the argument that history is not binding does not have clear implications. For example, it could be used to argue that beta (the rate of drag on innovation) is much smaller than in the past or that substitution will be greater in the future. This would imply that limits to growth are even further off that we suggest.

The usual response to these concerns is either using history as a guide (which is typically done) or using physical theory to guide predictions. We choose the former approach – to illustrate what might be possible. The latter is less useful. There are no obvious physical limits to substitution, even as there are physical limits to specific resources. The reason is that physics has little to tell us about what humans will want to consume, other than food and basic shelter.

More incidental comments

In the current state of affairs, the paragraph on lines 25 to 47 is a purely speculative and useless detour. I do not see the point of alluding to it. In any case, it does not contribute to ``mediating the dispute" that is the main focus of the paper.

Lines 25-47 in the submitted paper contained two paragraphs, one on improvements in productivity, and one explaining that the considerations in this paper will be limited to an Earth-bound civilization. The paragraph on improvements in productivity is necessary in the introduction as it is one of the main responses to the limits to growth literature and an idea that we further develop in this paper. The paragraph providing the caveat that we consider an Earth-bound civilization is necessary because the limits to growth arguments clearly break down for an extremely long time if it is relaxed. Although the limits to growth literature implicitly assumes an Earth-bound civilization, this assumption is typically not clearly stated. For the sake of rigor it is essential that this assumption be stated clearly in the introduction with some gesture toward its implications.

Lines 80 and 81: what does the acronym SI mean?

We have changed this to: "The Supplemental Information (SI) explains…" (line 80).

Line 188: Murphy's assumption is made for the sake of illustration as a "what if" exercise. Contrary to what the authors seem to suggest, it is not an assumption that he considers to be true (in his article, see the comment in the second paragraph below equation 2).

You are correct.

First, we should note that, while Murphy says CO2 emissions are potentially a bigger threat than waste heat, his paper focuses on and models waste heat. In order to engage in a rigorous analysis of limits to growth, we need to start with a rigorous argument or model. Murphy sets forth a rigorous model of waste heat, so that is what we engage

with. Had he set forth a model of greenhouse warming, we would have engaged that. Surely others have modeled greenhouse warming, but Murphy is an important, recent contribution to the limits to growth debate in a prominent journal.

Second, we changed the text to note that, while we focus on waste heat, it is for illustration. Moreover, our discussion of waste heat enables arguments that would be qualitatively similar to arguments that would be appropriate in a discussion of other limits, such as global warming. On line 65 we added:

> "In our discussion, we examine limits to growth other than waste heat and implications of our analysis for policy. There may be other limits to growth, but the discussion of when those limits will bind growth will be qualitatively similar to the discussion of limits due to waste heat. Ultimately, we conclude that limits certainly exist, but more research is required to determine when they will bind."

Line 136: the authors state that the units of variable W are

We have revised this sentence as follows (now line138): "...where W stands for Watts (kg m^2 s^{-3})..."

The intuitive reasoning leading to equation (4) could be made more explicit.

We have added the following to the sentence above Eq. (4) (line 209): "...via consideration of planetary energy balance…"

Computations underlying Figures 2 and 3: the authors could be more explicit about the way they compute g_Y and g_K.

We clarify our methods on lines 312-325. We also added the following explanation on line 356 (which overlaps a bit with the legend to Figure 2.)

> Forecasts start at 2019 levels of working population, power consumption and TFP (see \nameref{tab:values2019}). Forecasts assume that production is governed by a Cobb-Douglas function (\eqref{eq:CD}) with factor shares estimated using data from 1965-2019 (see \nameref{tab:growthRates}); that TFP levels are governed by equation \eqref{eq:A_t}, TFP growth by equation \eqref{eq:g_A}, power by equation \eqref{eq:P_t}, and power growth is derived from the time derivative of equation \eqref{eq:P_t}; and that output, working population and growth rates of factors other than power follow historical values from 1965-2018 (see \nameref{tab:growthRates}).

The legend to Figure 3 explains how Figure 3 is derived.

Line 387 about the U-shaped curve on panel F of figure 3: the authors consider this shape as particularly interesting. I think that the right-hand part of the U-shape is a direct consequence of the assumption they make on the law of motion of technological progress.

That is correct. However, note that the assumptions on beta and lambda are not arbitrary: they are the returns to population and drag on technological development that match historical rates of power consumption under the assumption we make about the parametric shape of g_A in eq. (7). We clarify this in lines 407-409.

Supporting information from lines 553 to 603. These two sections (in particular the second one) are really elementary. I don' t them think they are very useful here.

With all due respect, we wish to retain these sections. This is an interdisciplinary paper targeted at natural scientists and economists. While the economic terms we use may be obvious to economists, in our presentation of this paper to individual scientists, we found they were confused by our economic terminology. These 2 appendix (SI) sections can help natural scientists understand what specifically we mean by dollars and why we use dollars as a unit, and what we mean by output, income and welfare.
