# Supplementary material for: Revisiting the physical limits to economic growth, with a focus on the waste heat limit
Source: PLoS One. 2025 Mar 13;20(3):e0319217. doi: 10.1371/journal.pone.0319217 (PMC11906051; doi:10.1371/journal.pone.0319217)
Supplement: Attachment — Submitted filename: PLOS Review Response 2.pdf [file pone.0319217.s005.pdf]

Dear Dr. Boulanouar,

We thank you for your time and help with this paper, which we think has been improved by the review process. In order to respond to the current round of comments by the second reviewer, we need to take a step back from the specifics of the current paper and discuss the philosophy and intent of the review process based on experience and insight we have gained from writing approximately two hundred peer-reviewed journal articles and reviewing for more than one hundred journals. The primary purpose of the review process is to identify technical errors to prevent confusion in the literature. In the course of things it is common that good-faith practitioners will disagree on the interpretation, synthesis, and presentation of an assemblage of data and theoretical calculations. In such cases the disagreement is supposed to play out in the open in published work and comments on published work, not in the review process. It appears that we have reached such a stage with this paper. We value and appreciate the reviewer's perspective, and think it has merit; however, after considering the preponderance of evidence, while acknowledging that others might think differently, we simply have arrived at different conclusions than the reviewer has. For these reasons we do not think it is appropriate or necessary to modify the paper further. We respond in detail to the reviewer's comments below and we encourage the reviewer to write a response to this paper in order to criticize aspects of our paper he or she disagrees with and get his or her important perspective out in the open literature.

Sincerely,
Dorian S. Abbot and Anup Malani

Reviewer #2: Thank you for the stimulating intellectual exchange. We appreciate your feedback and think it has helped improve our paper. We would love to read your ideas and thoughts in this area fully developed in a peer-reviewed paper.

a) If I am not mistaken, the authors have not made any comment on my remark about their (optimistic) assumption that the innovation process is not resource consuming (here power consuming). It is not incidental: there are various contributions to the economic growth literature showing that global productivity gains could be slowed down by stronger environmental constraints, something which is absent from the innovation process described by equation (7).

Note that we do not assume that the innovation process is not energy consuming. Instead, our assumption is that the innovation process is not <u>net</u> energy consuming. This is validated by the increasing productivity of power over time in Figure 1.

It should also be noted that the reviewer does not provide any evidence that the process of innovation – as opposed to the process of production – consumes power on par with the level of production. We do assume that production is energy consuming, i.e., Y is a function of P, even if growth in A is not. (Perhaps the reviewer is thinking of AI, which requires power for learning. But this is a recent feature of innovation. Many other innovations require less power than manufacturing of the output from innovation. For example, manufacturing MRNA vaccines likely took much more power than the development of such vaccines. Moreover, we still do not know the total power consumption by uses of AI.)

A secondary point that the reviewer makes, not dependent on power per se, is that there may be productivity gains slowed down by environmental constraints. We address variation in the rates of productivity growth in our sensitivity analysis. For example, Figure 3 examines what happens as power plays a more important role in production (panel B), as innovative returns from population growth decline (panel C), and as the cost of incremental innovation rises (panel D).

Moreover, we address alternative constraints – other than power – in our section on substitution. There we show that there has been a great deal of substitution of inputs, suggesting the potential of addressing specific resource constraints by either shifting to other inputs for the same product or by shifting to consumption of other products which reduce consumption of specific constrained inputs. We believe the reviewer acknowledges this in her/his original report ("the section entitled `Growth due to substitution to other resources' … implicitly aims at answering to my previous point of criticism").

b) The section entitled 'Growth due to substitution to other resources' remains essentially unchanged. Therefore, quite logically, my point of view about this section and the status of its conclusion remains essentially the same as in my first report.

We have already modified the paper to justify the inclusion of this section and defended this in our previous response. In our opinion, the section fits and belongs in the paper. The reviewer wants this section removed, then the title modified to reflect a smaller range of investigation (next comment). That's a fine opinion for the reviewer to have, but we don't share that opinion, and it's our paper. We are not making any obviously incorrect arguments, just arguments the reviewer disagrees with. This is a dispute that should occur in the open, not in a review process. We encourage the reviewer to write a rebuttal paper or comment on our paper.

1) The title of the article should make explicit the perspective according to which the physical limits to growth are revisited. The concept of "waste heat limit" should therefore appear in the title because it is in reference to this concept that the [main] results of the paper are obtained.

Murphy's 2022 paper is titled "Limits to economic growth" even though it primarily considers waste heat. We note in the abstract that we are, "focusing especially on the waste heat limit." We do consider other factors, such as substitution to other resources (see last comments). Given all of this, it is appropriate for us to keep the title, "Revisiting the physical limits to economic growth."

2) If the authors quote the 1972 report (The Limits to Growth) several times, they cite relatively few later contributions to the literature on environmental limits to economic growth. In particular, they make almost no reference to contributions that could be classified as ``mathematical ecological economics". Beyond Murphy, they now mention Meran and Baumgärtner, whom I had mentioned in my frist report, but no one else. Even if this field is not dominant in Economics, it is at the very heart of the dispute that the authors want to contribute to resolve. I think it would therefore be justified to make more reference to it. Alternatively, it would be better not to present the article as an attempt at "mediating a dispute" but to focus on the goal of revisiting Murphy's analysis.

In writing the introduction to a paper the proper balance must be struck between discussing everything that has ever been done and highlighting the most important contributions in an area. We think we have hit that balance well by foregrounding the original literature motivating the limits to growth idea including the 1972 report and Erlich's famous work, as well as the recent article by Murphy in a prominent physics journal. This balance quickly brings the reader up to speed with all of the critical arguments necessary for understanding our contribution, and points to the most important papers that should be studied if the reader wishes to go deeper into the subject. The reviewer disagrees with our choice of sources, which is fine, and often happens when different people write the same subject. We encourage the reviewer to write his or her own paper and cite the sources he or she thinks are most appropriate in it.

3) The authors should avoid statements that could seem to be contradictory, even to a well-meaning reader. In the abstract for instance, the authors outline a "remarkable parameter sensitivity: across a plausible range of parameters waste heat never constrains economic growth or does so in a few hundred years". But a few lines below,

they end the abstract by writing "our work highlights the potential of an optimistic future for humanity in which economic growth continues on a millennial perspective". From the former careful sentence to the latter assertive one, it seems to be a big gap. At the very least, the authors should state the conditions under which their last assertion could be met.

These sentences are not contradictory. *Because* of the parameter sensitivity there is the *potential* for an optimistic future for humanity in which economic growth continues on a millennial perspective.

Other comments:

As far as the "Supporting information" section is concerned, the authors explain in their letter why they wish to keep this section. If I follow their point, I must conclude that there is then a strange asymmetry in the paper: basic economic concepts are explained in detail while (not so basic) concepts of physics are not. The authors seem to assume that economists know more about Physics than natural scientists know about Economics. I am not as sure as they are...

We thank the reviewer for sharing this perspective and encourage the reviewer to write his or her own paper containing what he or she thinks is the proper balance of concepts from physics and economics.

Line 525: the authors write: "When business is conducted and experiences are provided virtually, GDP can be increased even as energy usage is decreased". Virtually maybe but if I take a global (Worldwide) perspective, this has never been the case (at least until 2020). At the level of some countries, an absolute decoupling has recently been observed. But it is not the case at the World level (which is, to my eyes, the relevant one for the issue at stake here): until now (or at least until 2020 since I have not checked since then), World economic growth has always gone hand in hand with higher energy consumption (see e.g. bp energy outlook).

This paragraph is doing theoretical work in the discussion section, where free-ranging discussion of implications, limitations, and associations of a paper are encouraged. The paragraph is not meant to be a description of historical trends, although, as the reviewer notes, the particular sentence highlighted describes a phenomenon that has already been observed in individual countries.

In addition, experiences of less developed countries often mimic those of more developed countries as the latter develop further. So, for example, we see growth in online purchasing and the rise of delivery services–-and associated energy consumption changes–in India over time. Indeed, it has been noted that less developed countries achieve technological gains of developed countries at lower levels of real income, in part due to technology transfer. These

are reasons to suspect what is now a phenomenon in more developed countries will spread to less developed ones in due time.
